# Extracellular Vesicles in Type 1 Diabetes: A Versatile Tool

**DOI:** 10.3390/bioengineering9030105

**Published:** 2022-03-04

**Authors:** Caitlin N. Suire, Mangesh D. Hade

**Affiliations:** Department of Biomedical Sciences, College of Medicine, Florida State University, Tallahassee, FL 32306, USA; caitlin.suire@med.fsu.edu

**Keywords:** type 1 diabetes, T1DM, pancreas transplant, islet β-cell transplant, therapy, extracellular vesicles, EVs, exosomes, biomarkers, microRNA

## Abstract

Type 1 diabetes is a chronic autoimmune disease affecting nearly 35 million people. This disease develops as T-cells continually attack the β-cells of the islets of Langerhans in the pancreas, which leads to β-cell death, and steadily decreasing secretion of insulin. Lowered levels of insulin minimize the uptake of glucose into cells, thus putting the body in a hyperglycemic state. Despite significant progress in the understanding of the pathophysiology of this disease, there is a need for novel developments in the diagnostics and management of type 1 diabetes. Extracellular vesicles (EVs) are lipid-bound nanoparticles that contain diverse content from their cell of origin and can be used as a biomarker for both the onset of diabetes and transplantation rejection. Furthermore, vesicles can be loaded with therapeutic cargo and delivered in conjunction with a transplant to increase cell survival and long-term outcomes. Crucially, several studies have linked EVs and their cargos to the progression of type 1 diabetes. As a result, gaining a better understanding of EVs would help researchers better comprehend the utility of EVs in regulating and understanding type 1 diabetes. EVs are a composition of biologically active components such as nucleic acids, proteins, metabolites, and lipids that can be transported to particular cells/tissues through the blood system. Through their varied content, EVs can serve as a flexible aid in the diagnosis and management of type 1 diabetes. In this review, we provide an overview of existing knowledge about EVs. We also cover the role of EVs in the pathogenesis, detection, and treatment of type 1 diabetes and the function of EVs in pancreas and islet β-cell transplantation.

## 1. Introduction

Diabetes mellitus is a lifelong, incurable disease in which the production or response to insulin is dysregulated. Diabetes diagnoses are divided into separate categories based on their mechanism, with the two most commonly discussed groups being type 1 diabetes mellitus (T1DM) and type 2 diabetes mellitus (T2DM). Approximately 90% of diabetes cases can be considered T2DM, which occurs when cells do not respond appropriately to circulating insulin (called insulin resistance) and thus need higher insulin levels to regulate sugar processing. Doctors often recommend an initial intervention of modified diet and exercise to increase insulin sensitivity. As needed, T2DM patients are given external insulin to decrease blood sugar levels.

T1DM accounts for the remaining 10% of diabetes cases and is caused by the immune-mediated T-cell attack on insulin-producing β-cells in the pancreatic islets. This produces a deficiency in insulin, hyperglycemia (high glucose levels), and changes to the metabolism of proteins and lipids [1]. In total, 34.2 million patients are currently living with T1DM globally, a number that has tripled in the last decade. An estimated 500,000 of these patients are less than 15 years of age, giving T1DM its common moniker of “juvenile diabetes” [2,3,4]. Treatment of T1DM is more complex than T2DM, as it cannot be regulated solely by diet and exercise, is regularly accompanied by severe complications, and has extreme variability due to the diverse ages of diagnosis. Primary treatment options for T1DM include continuous exogenous insulin and immunosuppressive drugs, which ameliorate the primary pathology of T1DM. Globally, one in every two patients are unable to afford or lack access to the T1DM therapies they need [5]. The number of diabetes diagnoses is steadily rising, as, by 2045, the number of people with diabetes is estimated to reach 629 million, making diabetes management a critical target for social and economic stability [5,6]. Further, though the diagnosis of T1DM can take place using a number of measures, including common symptoms of frequent urination and fatigue, problems can arise with late or delayed diagnosis [2,7]. Therefore, due to regular complications, the anticipated rise in the number of diabetic patients, and the rising costs of insulin, there is a drastic need for a change in approach and alternative treatments for type 1 diabetes.

One potential option in the management of T1DM is the use of EVs. EVs are a heterogeneous population of membranous vesicles secreted from diverse cells that have assorted cargoes such as proteins, lipids, and RNAs that are representative of their parent cells. These small particles are shown to play essential roles in disease and offer an impactful insight into cellular communication during the challenge. This review covers current solutions in the treatment of T1DM, with a focus on pancreatic and islet transplantation and the potential benefits of EVs in T1DM diagnosis and management.

## 2. Extracellular Vesicles in T1DM

T1DM is a complicated disorder that results from an intricate interaction between environmental and genetic components, with the former involving many susceptibility genes. The majority of T1DM patients are dependent on lifelong insulin treatment, which can only alleviate symptoms rather than restore function. EVs have lately gained popularity as possible diagnostic tools that could lead to the discovery of new biomarkers and therapeutic targets. Additionally, EVs have a unique ability to deliver bioactive molecules to precise locations and so have significant therapeutic implications. The following sections summarize about existing knowledge of EVs in T1DM in terms of diagnostics and therapy, as well as emerging EV based therapies.

## 3. Extracellular Vesicles

**Figure 1:**
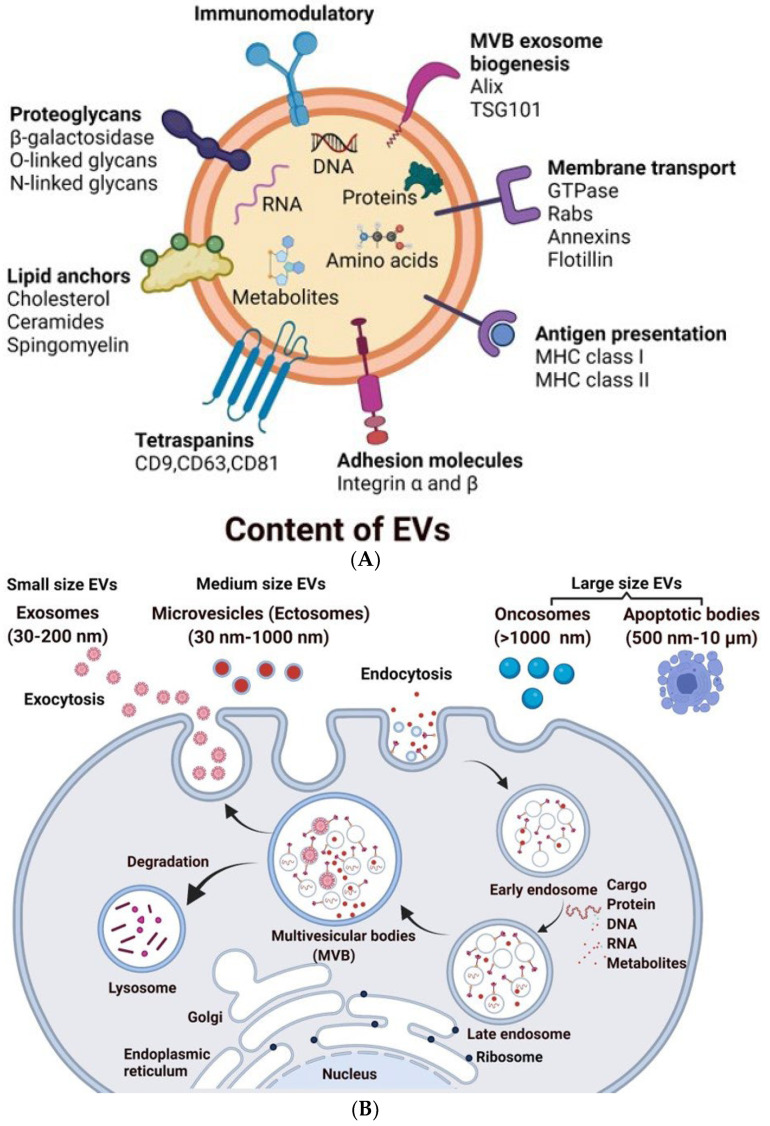
Schematic representation of the biogenesis of EVs: (**A**) Composition of EVs: EVs consist of cargo made up of bioactive molecules such as proteins, nucleotides, secondary metabolites, and lipids. Heat shock proteins (Hsp90 and Hsp70), Tetraspanins (CD63, CD9, and CD81), cytoskeletal proteins (Fibronectin and Actin), viral proteins (Tsg101), and enzymes are examples of proteins. EVs also contains DNA and RNA. (**B**) During endosomal maturation, multivesicular bodies (MVBs) develop, and exosomes are released when the MVBs fuse with the plasma membrane. Microvesicles, on the other hand, are derived directly via cell membrane budding and fission. Apoptotic bodies are formed by the death of apoptotic cells.

EVs are endogenous lipid bilayer vesicles that are secreted by the majority of cell types and are detected in virtually all bodily fluids [8,9,10,11]. EVs were thought to be a result of cells secreting and eliminating garbage [12,13,14], but research has determined that EVs are released into the surrounding environment by parent cells to execute a range of biological activities, including cell signaling and genetic material exchange (Figure 1A) [15,16,17,18]. This review gives a brief introduction to EVs; for more specific detailed information about the biological roles of EVs, cell biology, or cell to cell communication, readers are encouraged to refer to the excellent review articles by Ramis et al., 2020, and Kalluri et al., 2020 [19,20].

### 3.1. Classification and Origin of EVs

EVs are heterogeneous, ranging from 30 nm to 10 µm in diameter and encompass several types of vesicles, including but not limited to apoptotic bodies, which are large vesicles released from cells undergoing apoptosis; microvesicles, which are released from an evagination of the plasma membrane; and exosomes, which are formed in multivesicular bodies (MVBs), that then fuse with the plasma membrane to release the vesicles into the extracellular fluid (Figure 1B, Table 1) [10,12,21,22].

The endosomal membrane pathway produces exosomes from a variety of cell types. Multiple endocytic bodies cluster together to create early endosomes after the plasma membrane invaginates to make endocytic vesicles. The invagination of early endosomes then progressed to the formation of multivesicular bodies. Finally, MVBs migrate and make contact with the plasma membrane in order to secrete vesicles into the extracellular environment, which are known as exosomes [23]. Exosome formation includes cargo sorting and releasing material, and the correct modulation of this process necessitates the coordination of many different proteins and can be modified by lysosomal status [24]. Exosome biogenesis requires subunits of endosomal sorting complex required for transport (ESCRT), which include ESCRT complexes 0 to III [25].

Exosome secretion is divided into two types: ESCRT-independent and ESCRT-dependent. The ESCRT-0 complex recruits proteins via ubiquitin or clathrin in the ESCRT-dependent pathway. ESCRT-I and ESCRT-III regulate the budding process, the production of ILVs, and the subsequent synthesis of exosomes. Syntenin, ALIX, and ESCRT-III are involved in the ESCRT-independent mechanism. Syntenin promotes proteins to form cargo clusters, and then ALIX and ESCRT-III govern the budding process, ILV development, and exosome formation. Protein engagement and cargo sorting can be accomplished by either ESCRT-independent mechanisms or ESCRT-dependent mechanisms [25,26].

**Table 1:** Classification of EVs [27,28].

Characteristics of EVs				
	Small-Sized EVs	Medium-Sized EVs	Large EVs	
	Exosomes (Nanovesicles)	Microvesicles (Ectosomes)	Oncosomes	Apoptotic Bodies
Origin	Endocytic pathway	Cell plasma membrane	Cell plasma membrane	Cell plasma membrane
Size	30–200 nm	200–1000 nm	>1000 nm	500–10 µm
Common markers	CD63, CD9, CD81, TSG101, flotillin, Alix, ESCRT-3	Integrins, Selectins, CD40 ligand, ARF6, VCAMP3	Annexin A1, Annexin A2, ARF2	Annexin V, Phosphatidylserine, Thrombospondin
Content of EVs	Secondary metabolites, proteins and nucleic acids (mRNA, miRNA and other non-coding RNAs), lipids	Secondary metabolites, lipids, proteins and nucleic acids (mRNA, miRNA and other non-coding RNAs)	Nuclear fractions, cell organelles, proteins, DNA, coding and non-coding RNA, lipids	Nuclear fractions, cell organelles, proteins, DNA, coding and non-coding RNA, lipids
Pathways	Stimuli, ESCRT, and tetraspanin dependent	Stimuli, Ca^2+^, cell-dependent	Apoptosis related	Apoptosis related
Functions	Intercellular communication	Intercellular communication	Intercellular communication	Phagocytosis

### 3.2. Composition of EVs

The contents of EVs represent the function and content of their parent cells, and as such, a variety of proteins, enzymes, transcription factors, lipids, extracellular matrix proteins, receptors, metabolites, and nucleic acids are present both inside and on the surface of an EV (Figure 1A) [9,12,18,21,22,29,30]. The cargo contents vary depending on the specific stimulus or disease conditions during EV formation. Cells consist of several thousands of lipid species, and using mass spectrometry (MS), it is now possible to quantify approximately 1000 lipids in a sample. EV membrane is made up entirely of lipids, and it is widely known that certain lipids are more abundant in EVs than in their parent cells. EVs derived from known cell types contain the composition of lipid classes, including cholesterol (CHOL), phosphatidylinositol (PI), glycosphingolipids, SM, phosphatidylcholine (PC), phosphatidylserine (PS), and phosphatidylethanolamine (PE). The lipid composition of EVs from body fluids includes sphingolipid and PC [31,32]. In EVs, a wide range of genetic material is detected. A large number of short RNAs are identified using various approaches, including next-generation sequencing. Y RNA, tRNA fragments, long and short non-coding RNA, vault RNA, and piwi-interacting RNA are all discovered in EVs, in addition to generally recognized RNA species such as mRNAs, miRNAs, and rRNAs. Specific miRNA and mRNA packaging into EVs may be aided by certain sequence motifs and posttranscriptional modifications in the 3′ end of miRNAs [33,34]. Recent investigations demonstrated that EXOs include both mitochondrial DNA (mtDNA) and fragmented chromosomal DNA [35,36]. EVs contain DNA ranging from 100 base pairs to 2.5 kilobase pairs [37]. Despite the strong evidence that EV-DNA is present in EVs, the DNA cargo’s functional importance is unknown. The protein content of EVs is related to the type of cell and the biogenesis mechanism in which they are produced. Tetraspanins CD81, CD37, CD63, CD53, and CD82 and major histocompatibility complex class II (MHC class II) are more abundant in EVs originating from the endolysosomal compartment. Alix and tumor susceptibility gene protein 101 (TSG101) are two auxiliary proteins required for the endosomal sorting complex required for the transport (ESCRT) pathway. EVs, regardless of cell type, contain ESCRT proteins, Alix, TSG101, and chaperones such as Hcs70 and Hsp90. Typical protein families present in EVs are given in Table 2 [38,39].

### 3.3. Technologies for EV Isolation

Blood, urine, breast milk, cerebrospinal fluid, tears, saliva and nasal secretions, ascites, and sperm are all found to contain EVs. Many breakthroughs in EV detection and separation techniques were made in recent decades. As a result, EV recovery, purity, sensitivity, and specificity were all improved (Table 3). It is crucial to establish that the isolated vesicles are EVs with no contaminating components before doing any functional study. Despite this, challenges in isolation approaches exist due to overlapping size ranges, small diameters, and comparable morphologies to other EVs.

There is no unanimous agreement on the ideal EV isolation strategy. Despite the fact that MVs and EVs are formed by independent pathways, due to their size overlap, most separation methods do not separate a pure population. Density gradient centrifugation, ultrafiltration, ultracentrifugation/differential centrifugation, polymer-based precipitation, immunoprecipitation, microfluidic device isolation, and size exclusion chromatography (SEC) are just a few of the EV separation technologies that are developed thus far [40].

## 4. Background in T1DM

### 4.1. Healthy Blood Sugar Regulation

**Figure 2:**
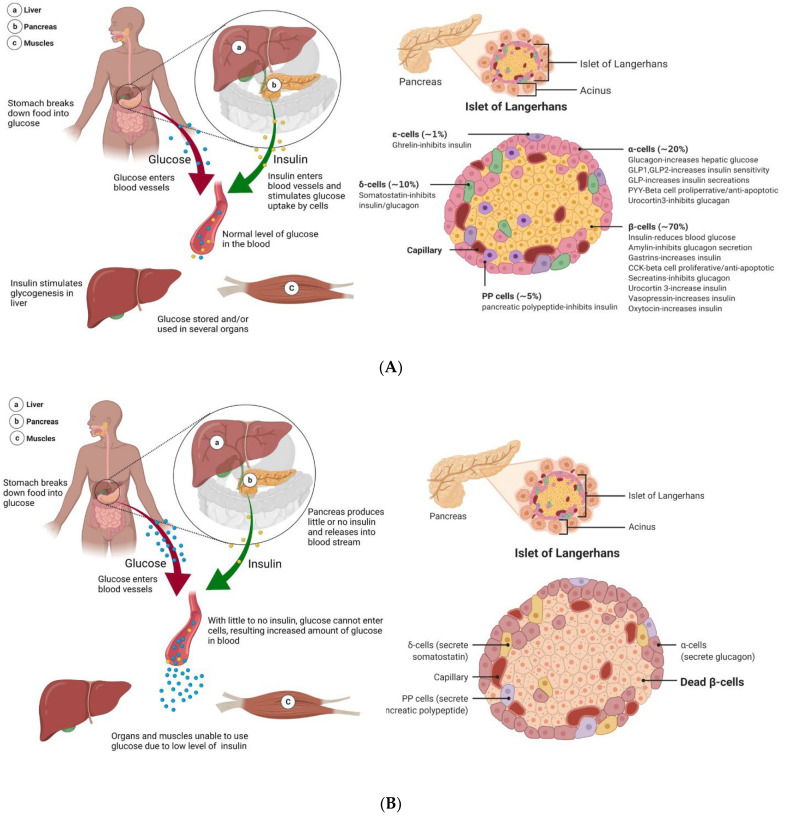
Processing of glucose in healthy and T1DM patients. (**A**) As food is ingested in a healthy system, it gets broken down into glucose, which is then released into the bloodstream. In response, the pancreas secretes insulin. Glucose is transported across the membrane via facilitated diffusion. As such, as insulin increases the glucose permeability of cells, this allows the uptake and utilization of glucose. The pancreas is made up of many important cell structures, including the islets of Langerhans. The islets are a low percentage of the total pancreas mass but include cells that are vital in glucose regulation. This includes β-cells, the primary component of islets, making up 65–80% of the total islet cell count. β-cells are responsible for the production and secretion of insulin and amylin. (**B**) In diabetic patients, as food is digested and converted into glucose to be released into the bloodstream, the normal corresponding insulin response is lacking. This is because the pancreas is no longer producing enough insulin to enable the uptake and utilization of glucose, leading to a state of hyperglycemia. In T1DM, a large number of β-cells in the pancreas are killed due to autoimmune instigated T-cell attacks. Though some β-cells may remain, the pancreas is no longer capable of insulin independence.

Diabetes is a disease in which metabolic processing and energy regulation malfunction. Energy in the body is provided through an intricate balance of food intake, breakdown, and uptake. In a healthy, non-diabetic system, the carbohydrates, lipids, and proteins from food are broken down into glucose, which is then delivered to cells via facilitated diffusion to be used for energy. Insulin is the vital gatekeeper in this process, regulating blood glucose levels (also known as blood sugar) by triggering cells to take up glucose from the blood (Figure 2A) [41,42,43]. This increase in membrane permeability is thought to be a result of the translocation of glucose receptors to the cell membrane [42,43]. The pancreas plays a key role in this process, as it aids in the digestion, absorption, utilization, and storage of energy substrates [41,44]. The pancreas is located close to other important organs in metabolism, lying below the stomach and extending from the spleen to the duodenum [45]. The pancreas is structurally distinct, containing cells that perform exocrine (nutrient digestion) and endocrine functions (glucose homeostasis) [44,45]. Though endocrine cells, which make up clusters called the islets of Langerhans (or pancreatic islets), are vital in the regulation of blood sugar, they make up only 1–2% of the adult pancreas [7,44,45,46]. Each islet has a number of distinct cell types, which produce hormones that play different roles in blood sugar maintenance. This includes α-cells and δ-cells, which produce glucagon (which has a role opposite to that of insulin, raising blood glucose levels) and somatostatin (which regulates the secretion of other hormones from the pancreas), respectively (Figure 2A) [44,45]. β-cells, which make up 65 to 80% of islets, produce insulin and amylin at a 100:1 ratio, which collaboratively works to prevent blood sugar spikes by triggering the uptake of glucose into cells and promoting satiety [44,45,46,47]. Each β-cell produces up to 1 million insulin molecules/minute (a number that is further elevated in response to eating) [7,46]. Insulin is considered a “key” in sugar regulation, allowing cells to uptake glucose by increasing the permeability of plasma membrane to glucose, inducing the conversion of glucose to glycogen in the liver, and promoting the production of fatty acids and proteins [46]. Together, the cells of the pancreas and the hormones secreted regulate blood sugar, with the average, optimal 24 h glucose levels of 99 ± 7 mg/dL [48]. In non-optimal, hypoglycemic conditions, blood sugar levels are below ideal levels, triggering the secretion of glucagon from α-cells, which promotes the breakdown of glycogen stored in the liver to glucose [46]. When the body has high blood glucose content, called a hyperglycemic state, β-cells release insulin and amylin to reduce the blood sugar levels and decrease food intake [46]. This system malfunctions in T1DM causing insulin levels drop, and blood sugar levels spike as cells are unable to take up glucose.

### 4.2. Mechanisms in T1DM

Type 1 diabetes is a multifaceted disorder produced by a complex interplay of environmental and genetic variables, the former of which includes several susceptibility genes. The discovery of genes that confer vulnerability to T1DM would help to clarify pathogenetic processes in the establishment and progression of the disease, paving the way for the development of effective approaches for disease prevention and management. Genome-wide linkage analysis and genome-wide association studies (GWAS) and genome-wide linkage analysis have recently contributed significantly to our understanding of the role of genetics in the onset and progression of T1DM. Nearly 60 risk regions all over the human genome were found by GWAS, which is designated by single nucleotide polymorphisms that confer genetic susceptibility to T1DM [49,50]. Several studies claimed that 50% of the risk factors for T1DM are genetic [51]. When researchers first began investigating the causes of T1DM, genetic risk factors were focused on as the sole trigger, as susceptibility to developing T1DM is influenced by carrying specific high-risk haplotypes of class II human leukocyte antigens (HLA), such as HLA-DR (DR3/4) and HLA-DQ (DQ8) [4,52,53]. Over 50 non-human leukocyte antigen (HLA) regions and HLA genes were discovered in T1DM using genomic screening [51]. HLA-DQ and HLA-DR, in particular, are closely linked to T1DM. Haplotypes of class II molecules antigens can substantially increase or decrease the binding properties of associated autoantigens, resulting in T1DM development [54,55]. The impact of HLA on T1DM risk is supported by a twin concordance rate of up to 70% and 8–10% of risk shared between siblings [56]. HLA genetic susceptibility contributes to the loss of tolerance of β-cells antigens due to T-cell mediated islet cell death, insulin deficiency, and elevated blood glucose levels, suggesting that these genes operate at the β-cell level and play a crucial role in the pathogenesis of T1DM [3,57]. Despite initial thoughts, genetic predisposition alone cannot explain the incidence of T1DM, as recent work has identified that HLA loci account for 50% of familial T1DM [4,6,53,56,58]. Now, T1DM is considered an intricate interaction and balance between the genome, metabolism, and gut microbiome diversity [4,6,53,59,60,61]. There are also a number of environmental influences, including diet, vitamin D deficiency, infections, pollutants, and oxidative stress, associated with the increasing presentation of type 1 diabetes, with β-cell stress potentially providing a mechanistic underpinning [6,7,56,59,60]. In addition, comorbidities are often found in those diagnosed with T1DM, as the majority of T1DM patients (60%) are obese or overweight, while many others have hyperlipidemia and hypertension [62]. Comorbidities can occur as complications of T1DM, with individuals with poorly regulated T1DM having a 10 times higher chance of dying from cardiovascular disease than healthy individuals [4]. Though T1DM is slightly more common in males than females, females are more likely to experience complications of T1DM such as hypoglycemia, vascular events, and premature lethality in response to T1DM than males [6,62,63].

The mechanisms of T1DM begin well before full diabetes, as T-cells attack insulin-producing β-cells (Figure 2B) [6,7,53]. During this prediabetes phase, the pancreas can maintain normal glucose processing. However, the amount of insulin that can be secreted in the body is reliant both upon the absolute number of β-cells and β-cell function [46]. As the immune-mediated destruction of β-cells continues, this puts increasing demand and thus limits the function of the remaining β-cells. The body enters a state of hyperglycemia, with excessive levels of unused glucose in the bloodstream. In a healthy system, protective immune activated inflammation is terminated after a threat is eliminated, but the autoimmunity of T1DM leads to chronic inflammation, further promoting disease progression, β-cell apoptosis, and influencing insulin resistance [64,65]. Though some patients suffer from a complete loss of functional β-cells, measurable levels of C-peptide, which is the biologically inactive peptide formed when β-cells convert proinsulin to insulin, indicate that some T1DM patients can produce insulin; nevertheless, the functional β-cell mass is still decreased to the level of insulin-dependency [2,7]. In the 100 years since the discovery of insulin, knowledge of the T1DM pathology process, improved treatment options, and novel diagnostic measures were developed.

### 4.3. Role of EVs in Pancreas and Islet β-Cell Transplantation

**Figure 3:**
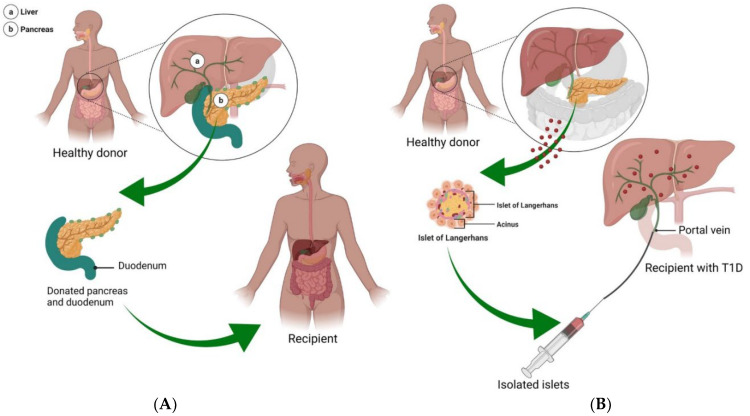
Pancreas and islet cell transplantation. (**A**) In T1DM, many of the islet β-cells die, limiting insulin production and leading to a number of life-long complications. The only near-cure treatment strategy is the transplantation of a pancreas from a healthy donor. In this situation, a pancreas with a small portion of the duodenum attached is inserted into the T1DM patient. With this approach, recipients are able to achieve up to 5 years of insulin independence. (**B**) Considered an experimental procedure, delivery of islet cells to T1DM patients offers a less invasive and lower risk treatment option, in which islet cells from 1 to 3 healthy donors are isolated and delivered to the pancreas via the portal vein.

The continuous glucose monitoring systems, unfortunately, are not capable of rapid responses to glycemic variability as might be produced by illness, stress, or a simple unannounced meal [2]. Moreover, though insulin administration offers some regulatory abilities, many patients develop chronic complications or become disabled by refractory hypoglycemia [66,67]. An alternative treatment option is the transplantation of the pancreas or islet β-cells to prevent or slow the complications and progression of T1DM, improving longevity and quality of life (Figure 3A). Though pancreas transplantations continued to evolve and develop over time (for the full record, see [66]), the number of pancreas transplants are dropping. This is due to inherent surgery risk and improvements in other treatment options, with a 20% decrease in transplants in the USA between 2005 and 2014, transplants peaking in 2004 [44,68]. Transplantations as treatments for T1DM are generally only considered in patients who exhibit a history of hard to control diabetes, defined as ≥1 episode of severe hypoglycemia per year, frequent and severe metabolic or microvascular complications, and problems with exogenous insulin therapy [69,70,71,72]. As such, pancreas transplantations are typically only performed 20 years after diabetes diagnosis [69]. Transplanting a pancreas can fully replace the β-cell mass and as such, is considered the only “near-cure” for T1DM, while transplanting islet β-cells can only partially restore the population of insulin-secreting cells needed for insulin and glucose regulation [66]. The first documented pancreas transplants were performed in the 1960s–1970s on uremic diabetic patients in combination with a kidney transplant [44,48,66]. Early surgeries found that transplanting a pancreas alone led to a high rate of organ rejection, while cases in which a pancreas and a kidney were simultaneously transplanted had minimal organ failure (though were susceptible to numerous other issues accompanying surgery such as thrombosis or infection) [66]. Recent reports indicate that this graft survival trend has continued, with 70% graft survival in patients with simultaneous pancreas and kidney transplant, and only ~50% for pancreas in isolation [2,44,68].

As an alternative to pancreas transplant, islet β-cell transplantation is a low-risk procedure, with donor islets infused into the liver via the portal vein (Figure 3B) [6,73]. Though islet transplants are practiced internationally, this procedure is still considered experimental in the USA [74]. Though islet transplantation does not replace the entire β-cell mass, it is less invasive than a pancreas transplant while still providing the potential to restore normal glycemic function, re-establish hypoglycemia awareness, and minimize severe hypoglycemic responses [2,67]. Islet transplants were first attempted in the 1970s, with very low success rates [71]. The first fruitful islet transplantations occurred in the late 1980s and 1990s, with the ability to consistently establish endogenous insulin secretion via transplant taking place in the 2000s [48]. The principal indications for β-cell replacement therapy are to treat a small subset of insulin-dependent T1DM and T2DM patients with severe or uncontrollable hypoglycemia [6,75]. An estimated 50% of transplant recipients maintain insulin independence one to two years post islet transplant [45,73,76]. A recent approach in islet delivery is to encapsulate islets to protect them from the immunological environment, allowing greater survival and function [2]. Islet cells may be transplanted in combination with hormones or growth factors such as glucagon-like peptide-1 to augment insulin secretion and inhibit glucagon release [56,77,78].

There are several pieces of evidence that indicate that EVs may increase the survival and efficacy of pancreatic islet transplantation [79]. The communication between β-cells and endothelial cells is imperative for islet transplantation because it helps the body re-grow new blood vessels. Figliolini et al. showed that EVs derived from human islets could be taken up by endothelial cells inside the islets and help with angiogenesis and engraftment, according to in vitro experiments [80]. Poor graft vascularization limits the success of islet transplantation. Cantaluppi et al. investigated that EVs secreted by endothelial progenitor cells promoted human islet vascularization. EVs stimulated migration, proliferation, cell survival, and organization in vessel-like structures in islet endothelial cells [81]. EVs derived from endothelial progenitor cells increased insulin secretion, survival, and angiogenesis of islets implanted in severe combined immunodeficiency (SCID) mice after incorporation into islet endothelium and β-cells [81]. EVs can facilitate islet transplantation through immunomodulatory effects in addition to enhancing revascularization. Human bone marrow MSCs and their EVs have been shown to be capable of suppressing immunological responses and delivering small RNAs. As a result, they may be able to improve islet transplantation by delivering small RNAs that enhance islet function while preventing immunological rejection. EVs produced from MSCs are shown to promote islet transplantation by promoting regulatory T cell function and suppressing peripheral blood mononuclear cell proliferation, according to a study conducted by Wen et al. [82]. The therapeutic cargo contained within EVs must be appropriately studied for health and safety issues. In conclusion, exosomes may portray an enthralling therapy in the context of islet transplantation, not only for enhancing revascularization but also for stimulating graft tolerance.

### 4.4. EVs and T1DM Pathogenesis

T1DM is a complex, multifactorial disease that is heavily influenced by cross-talk between metabolically active tissues. EVs are thought to be a major mediator of cellular communication and have been found to be increasingly important in disease pathogenesis, as exposure of materials from cells in crisis affects the survival of recipient cells [38,83,84]. For example, EVs from obese or diabetic mouse models can induce insulin resistance and glucose intolerance, while EVs from healthy lean mice can reverse these symptoms [85,86]. EVs are shown to contain hormones and autoantigens that have a key role in insulin sensitivity and the initiation of the strong, detrimental autoimmune response in T1DM, including insulin receptor substrate 1, GAD65, IA-2, and proinsulin [87,88,89,90,91,92]. The injection of EVs containing autoantigens was found to increase cytokine release, stimulate Toll-like receptors, and facilitate graft rejection [90,93]. EVs containing high levels of autoantigens are released as the pancreas undergoes excess stress, as increased levels of glucose or treatment with proinflammatory cytokines were found to drastically increase EV secretion [87,89,91]. Interestingly, macrophage-derived EVs are able to activate memory T-cells [94]. In a similar fashion, EVs isolated from insulinomas can induce the secretion of inflammatory cytokines in diabetes mouse models through the MyD88-dependent pathway, which is downstream of inflammatory signals of Toll-like receptors and interleukin-1. Activation of MyD88 can trigger T-cell proliferation and activation, further stimulating detrimental autoimmune response in T1DM [88,95]. EVs also participate in a negative feedback loop of cytokine production, which further drives macrophage activation [96].

Micro-RNAs (miRNAs) play key regulatory functions in various aspects of blood sugar regulation, such as embryonic development in pancreatic islets, maintenance of α- and β-cell phenotypes, insulin secretion, and exocytosis [84]. The miRNA content of EVs can play a major role in propagating T1DM pathology. EVs secreted from cells exposed to cytokines led to apoptosis when delivered to β-cells, an effect that can be blocked through the inactivation of miRNA activity through inhibition of Ago2 (a component of the RISC complex that is essential for miRNA action) [83]. Increased EV expression of miR-21, miR-34a, miR-146a, and miR-29 as triggered by exposure to cytokines early in disease contributes to the cytokine-induced β-cell dysfunction [53]. The release of miR-375 and miR-148a-3p in EVs are associated with β-cell death [97]. Similarly, EV-contained miR-142-3p/5p and miR-155 lead to the upregulation of cytokines in pancreatic islets β-cells, with in vitro inhibition of these miRNAs able to reduce apoptosis rate in primary rat islet cells [90]. When splenocytes isolated from diabetes-prone mice are exposed to β-cell EVs, there is a miR-29b-dependent increase in the production and secretion of TNF-α [90]. Further understanding of how the contents of EVs are modified in disease states can give insight into the spread of inflammation and pathology in T1DM.

### 4.5. EVs as Novel Biomarkers in T1DM

Several lines of evidence suggest that many factors induce T1DM, including genetic predisposing factors, non-infectious environment agents, endogenous antigens, exogenous infectious pathogens, and physiological stress [4,6,56,59,60]. As such, the underlying autoimmune process of T1DM precedes the onset of clinical symptoms. Though the early stage of T1DM, called prediabetes, is asymptomatic, it provides an excellent advantage for early prognosis and prevention of disease [98]. Currently, susceptibility genes (high-risk HLA genes; HLA-DR (DR3/4) and HLA-DQ (DQ8)) and hallmark islet autoantibodies (GAD65, IA-2, insulin, and ZnT8) are the gold-standard approaches for the detection of T1DM. Notably, a clinical study has revealed that HLA typing identified only 10% of study subjects with high T1DM risk while the remaining 90% displayed no autoimmunity, and less than 50% of cases were identified by a combination of genetic markers [52,53]. Further, a major limitation of diagnostic autoantibodies is that they appear relatively late during disease progression when almost 90% of β-cells are lost [99,100]. Additionally, a subset of individuals who showed seropositivity for autoantibodies will not develop clinical diabetes [101]. Therefore, current diagnostic tools do not meet the need for early diagnosis, creating a great demand for the identification of a suitable biomarker for early risk prediction.

In recent years, several studies documented the potential utility of EV-based biomarkers in ailments such as liver disease, neurodegenerative diseases, and cardiac disorders [102,103,104,105,106,107]. EVs have easy accessibility from bodily fluids and molecular and structural stabilities, making them an excellent candidate for harboring biomarkers in clinical settings [108]. In the context of T1DM, EVs drawn from blood can offer a new understanding of the status of sugar regulation (Table 4). Evidence indicates that EVs secreted by human pancreatic insulin-producing β-cells may trigger and accelerate disease progression via stimulating autoimmune responses [88,109,110]. Studies also implicated that the severity of T1DM and its pathophysiological conditions, such as inflammation, can alter the yield, content, and composition of EVs released from β-cells, information that can be utilized in the discovery of biomarkers [47,83,108,111,112,113,114,115,116]. A study by Garcia-Contreas and colleagues found that T1DM patients had reduced plasma EV size and concentration compared to healthy controls [108]. A recent report of EV RNA profiling of human islets from T1DM patients revealed modified EV RNA expression in response to proinflammatory cytokine treatment. Treating islets with proinflammatory cytokines mimics the chronic inflammation found in T1DM and results in the differential expression of RNAs associated with necrosis, insulin secretion, calcium signaling, and apoptosis, emphasizing the toxic effect of inflammation on islet survival [117]. Krishnan and colleagues identified miR-155-5p as the most upregulated miRNA, while miR-4485 was the most downregulated [117]. Significantly, islet-derived EVs act as antigen carriers, including autoantibodies GAD65, ZnT8, and β-cell glucose transporter 2, which are released in response to cellular stress, and serve as markers of T1DM progression [87,95,97,114,118,119,120]. Importantly, islet EVs were found to specifically activate memory T-cell responses in diabetic patients, but not healthy controls [119].

MiRNAs are crucial in blood sugar regulation, and a diverse population of miRNAs can be found within EVs [84]. As such, EV miRNAs are considered attractive candidates for biomarkers to diagnose T1DM or determine the success of a treatment plan [84,121,122]. A number of miRNAs are consistently upregulated in T1DM, including miR-21-5p, miR-100-5p, miR-150-5p, miR-181a-5p, miR-210-5p, and miR-375, many of which can be found in EVs [53,122]. Work studying young, newly diagnosed T1DM patients identified distinct EV miRNAs modifications compared to healthy controls, with downregulated levels of miR-195 and miR-455, while miR185 was upregulated [115]. A study employed miRNA microarray and qPCR analysis to characterize plasma-derived EVs and identified a distinct miRNA profile found in patients with long-duration T1DM compared with healthy controls with seven differentially expressed miRNAs [123]. Distinctions in EV miRNA content were also found in children with urinary EV miR-424 and miR-218, and serum EV miR-21-5p was found to be elevated in children with T1DM compared to healthy controls [114,124,125]. Of vital importance, research has suggested that miRNAs such as miR-21-5p have detrimental impacts in T1DM and can be linked with β-cell inflammation and apoptosis [126,127,128,129]. Work studying young, newly diagnosed T1DM patients identified distinct EV miRNA modifications compared to healthy controls, with downregulated levels of miR-195 and miR-455, while miR185 was upregulated [115]. Importantly, certain EV miRNAs are elevated in distinct timeframes and conditions; for example, work within a mouse model of diabetes found that miR-375 was elevated two weeks before the onset of diabetes and was thought to be a specific marker of deteriorating β-cell health and function [53,83,84]. Studies reported elevated levels of β-cell miR-21-5p in EVs in response to inflammatory cytokines treatments, both in animal models of type 1 diabetes and in vitro, while total levels are decreased [95,126,127,128,129].

There are a number of complications that can occur in diabetic patients, including diabetic nephropathy and retinopathy, a kidney disease that and complication damaging the blood vessels in the eye, respectively. Recent studies found that EVs and the miRNA content within EVs can aid in the early diagnoses of these complications [84,85,130,131,132,133,134,135]. Barutta and colleagues determined that miRNA-145 within urinary EVs is elevated in diabetic patients with diabetic nephropathy [130], while Kalani et al. demonstrated that increased levels of Wilm’s tumor-1 protein within urinary EVs could predict a decline in renal function [131]. Further, peroxisome proliferator-activated receptor-γ within EVs increases with disease severity of diabetic retinopathy, while miR-222 is decreased [132]. Furthermore, certain populations of EVs have significantly elevated levels of creatine in response to hyperglycemic events in T1DM patients [133].

In transplantation, the primary target time frame for both biomarkers and therapeutics is immediately after the graft to prevent surgical complications and graft rejection [136]. In the instance of transplantation, the organ or cells release donor-specific EVs that are involved in immune recognition and often influence rejection or graft tolerance; in the case of graft rejection, there is a significant decrease in transplant islet EV signal, as well as changes in EV miRNA and proteomic profiles, displaying distinct biomarker potential [137,138,139]. Notably, miRNAs in EVs can be beneficial in identifying successful T1DM treatments, as in many cases of malfunctioning transplantations, there are distinct upregulated and dysregulated miRNA and protein profiles that can be identified in EVs up to 5 years post-transplantation [138,140]. Additionally, studies identified that islet endothelial cells (IECs) and β-cells secrete a subset of miRNAs in EVs that take part in cross-talk between IECs and β-cells, thus promoting angiogenesis, a process limited in T1DM and inflammatory conditions, and often a limiting factor in transplantation efficacy [80,81,82,83,140]. As a marker of vascularization, these miRNAs can identify early problems in transplantation. Time course studies found that EVs from islet cells contain varying miRNA in response to inflammation and hypoxia, with miR-29b-3p and miR-216a-5p released in EVs 6 h after hypoxia and cytokine exposure, while miR-375 and miR-148a-3p were secreted 24 h after exposure [91]. Importantly, these stress conditions replicate negative conditions found in diabetic mice following islet transplantation [91]. Cumulatively, this evidence suggests that EVs and EV miRNAs are promising biomarkers for early T1DM prognosis and development, as well as conditions following pancreas or islet transplant.

**Table 4:** Studies utilizing EVs as biomarkers in diabetes.

Study	Experimental Subject	Collected Specimen	EV Isolation	EV Size	MSC EV Characterization	Biomarkers	Important Finding from the Studies
[141]	Rat	Blood	UC	NA	WB, FC	eNOS and caveolin-1	Decreased levels of eNOS and overexpression of caveolin-1 may serve as a biomarker for vascular injury
[117]	Human	Human islet cell	UC	<150 nm	EM, WB	piRNAs, snoRNAs, tRNAs, and lncRNAs	EV miRNAs may consider as potential circulating biomarkers for T1DM
[139]	Human	Plasma samples	SEC, UC	NA	WB	Islet endocrine hormone proteins and mRNAs	Circulating transplant islet-specific EVs has the potential to be a diagnostic tool for recurrent autoimmune T1DM after islet transplantation
[137]	Mice and Human	Human plasma and urine samples	SEC, UC	30–200 nm	NTA, EM, WB	miRNA	EV miRNAs as biomarkers for monitoring immune rejection
[142]	Human	Urine samples	UC	NA	WB	Water channel aquaporins	Role of water channel aquaporins AQP5 and AQP2 as novel biomarkers to help in classifying the clinical stage of diabetic nephropathy
[133]	Human	Urine samples	UC	40–100 nm	EM, NTA	Urinary podocyte	Analysis of urinary podocyte MPs may act as an early biomarker of glomerular injury in uncomplicated T1DM
[143]	Human	Plasma samples	UC	NA	NA	Cytokines and angiogenic factors	EVs isolated from plasma shows upregulated levels of cytokines and angiogenic agents in diabetic patients.
[134]	Human	Urine samples	UC, Filtration	NA	WB	Cystatin B and altered protease profiles	Enhanced cystatin B and altered protease profiles in EVs isolated from urine may act as biomarkers of kidney dysfunction in T1DM
[144]	Rat and Human	Kidney tissue and Urine samples	UC	NA	WB	Regucalcin	Lower levels of urinary exosomal regucalcin may act as a biomarker of diabetic kidney disease
[130]	Human	Urine samples	UC	<100 nm	EM, NTA, WB	miRNAs	EV miR-145 may act as a biomarker of T1DM
[131]	Human	Urine samples	UC	NA	WB	WT1 protein	Elevated expression of EV WT1 protein may act as a biomarker of T1DM

### 4.6. Paracrine Effects of EVs on Islet Cells

As carriers of vital proteins, miRNAs, and other contents, EVs have natural therapeutic benefits in various diseases, including T1DM (Table 5) [145]. Research found that the administration of EVs can help preserve organs in donor transplantation and aid in minimizing autoimmunity and cytokine release, thus minimizing immune rejection, improving viability and long-term survival [63,82,145,146,147]. Specifically, EVs derived from mesenchymal stem cells (MSCs) were found to suppress the development of T-cells and slow disease development [63,114,145]. EVs naturally contain a number of valuable proteins and miRNAs but can also be used to deliver select cargoes, either through modification of the parent cells or through specific loading of therapeutic content [148,149]. Wen et al. found that cell modifications in which EVs delivered plasmids to silence Fas and miR-375 in human islets improved resilience against inflammatory cytokines and suppressed immune reaction [82]. Application of MSC EVs, through the delivery and upregulation of vascular endothelial growth factor (VEGF), promoted insulin secretion in islets, as well as downregulated pro-apoptotic genes, upregulated pro-survival factors, and increased angiogenesis, thus targeting primary causes of graft loss [80,150]. MSC-derived EVs were found to be protective in diseases, including T1DM, serving to block apoptosis, a major issue in graft survival, through the inhibition of the phosphorylation of p38 [151]. In a mouse model of diabetes induced using streptozotocin, the delivery of bone-marrow MSC-derived EVs aided in the regeneration of islet cells [147,152]. EVs from adipocyte-derived stem cells can promote angiogenesis and modulate the immune response, attenuating T1DM pathology [95]. Delivery of EVs derived from endothelial progenitor cells can enhance human islet vascularization, promoting insulin secretion and islet survival [81], and it is hypothesized that EV delivery early in T1DM may help restore a patient’s original islet cells [153]. Adipocyte-derived stem cell EVs modulate the immune response and promote proangiogenic properties, a vital aid in transplant survival [95]. EVs derived from bone-marrow-MSCs have shown the ability to ameliorate insulin resistance developed through aging and T2DM through the delivery of miR-29b [154].

In addition, EVs from a number of cell types, including MSCs, can have therapeutic impacts on the many complications that surround T1DM, including cognitive deficits, cardiac dysfunction, microvascular complications, and even transplantation rejection [155,156,157,158]. EVs loaded with miR-222 injected into rabbits with T1DM, and diabetic retinopathy showed attenuation of retinal degeneration [159]. Diabetic nephropathy was improved through the delivery of EVs derived from MSCs and adipose-derived stem cells by induction of autophagy [85,160,161] Though a number of studies regarding EV therapeutics use a heterogeneous population of EVs, a study by McGuinness and team (2016) found that microvesicles specifically stimulated long-term recovery and function in diabetic mice, improving glucose, insulin, and glucagon levels compared to the smaller EV population exosomes [162].

Finally, many pancreas and islet transplant recipients receive treatments to aid in successful transplantation that, if loaded into EVs, may be better protected and enhance impact. For example, IL-2 receptor antibodies to provide better early posttransplant protection of islets and coadministration of a TNF-α inhibitor can lead to a higher probability of insulin independence, two cargos that may benefit from protective EV delivery [67,78]. The overexpression and delivery of insulin-like growth factor 1 and protein 3 gamma not only enhance the proliferation of β-cells but demonstrate the ability to regenerate and restore β-cells [56]. The restoration and regeneration of pancreatic islet cells are regulated in part through EV interaction with the pancreatic and duodenal homeobox-1 pathway, which is vital in pancreatic cell differentiation [163,164]. Further, MSC-EVs at the time of islet transplantation are shown to improve islet functions through the delivery of necessary nutrients and thus transplant tolerance [147,165]. The delivery of EV-loaded cargos is not limited to T1DM. Glucagon-like peptide-1 is a hormone commonly used in T2DM to augment insulin secretion, aiding in glycemic index and insulin independence, can be loaded into EVs for better, more protected distribution [77].

**Table 5:** EVs as therapeutics in T1DM.

Study	MSC Source	MSC EV Isolation	EV Size	MSC EV Characterization	Model Species/Cells	Intervention(s), Route, and Dose	Important Finding from the Studies
[166]	Bone marrow-derived MSCs	NA	NA	NA	Rat model of T1DM	Tail vein injection	EV miR-145 secreted by bone morrow MSCs shows neurorestorative effects in diabetic rats with stroke
[167]	Human urine-derived stem cells	UC 30% sucrose/D2O cushion	50–100 nm	Flow cytometry, WB, NTA	Adult male Sprague Dawley (SD) rats	Weekly with 100 μg of EV dissolved in PBS to a final volume of 200 μL via the tail vein	Reduction of the urine volume and urinary microalbumin excretion; prevention of podocyte and tubular epithelial cell apoptosis in diabetic rats
[152]	Bone marrow-derived MSCs	UC	100 nm	FC, EM	Albino female rat (STZ-induced rat model of T1DM)	Intraperitoneal injection	EVs derived from MSCs showed therapeutic and regenerative effects upon the pancreatic islet cells
[168]	Human umbilical cord blood-derivedendothelial progenitor cells	UC	50–60 nm	Tunable resistive pulse sensing analysis, EM, WB, NTA	Adult male SD rats	Subcutaneous injection with EVs 2 × 1010 or 1 × 1011 particles, dissolved in 200 μL of PBS	Increased angiogenesis through Erk1/2 signaling
[169]	Human umbilical cord blood-derivedendothelial progenitor cells	UC	40 to 80 nm	EM, WB	Adult male SD rats	Subcutaneous injection at wound sites with 100 μL PBS or EPC-EVs (100 μg) around the wounds	Enhanced wound healing by regulating vascular endothelial cells function
[170]	Adipose derived-MSCs	ExoQuick	200 nm	EM, WB	10-week-old SD rats	Intraperitoneal injection	Adipose tissue-derived MSC EVs enhanced the erectile function in diabetic rats
[155]	Bone-marrow MSCs	UC	NA	EM, WB	Streptozotocin-Induced Diabetic Nude Mouse Model	Intracerebroventricular administration PKH-labeled EVs (5 µg in 10 µL aCSF)	Improvement of cognitive impairments by repairing damaged neurons and astrocytes
[171]	Cardiomyocytes overexpressing HSP70 MSCs	UC	50–60 nm	EM, WB, NTA	Streptozotocin-Induced Diabetic Nude Mouse Model	Intraperitonial injections	Enhancement of Hsp20 in cardiomyocytes can give protection in diabetic hearts via the release of EVs
[172]	Human bone marrow-derived MSCs	ExoQuick, Precipitation	NA	NA	Male C57BL/6J mice	Intravenous injections	Attenuation of renal fibrosis
[173]	Human fibrocytes	UC	50–100 nm	EM, WB, NTA, FC	Diabetic B6.Leprdb/db mice (11–12 weeks old)	200 μL of PBS containing 0, 5 or 50 μg EV; 40 μL subcutaneously injected around the wounds sites; 40 μL were directly applied to the wound beds	Enhanced proliferation and migration of diabetic keratinocyte; increased wound closure
[174]	Mouse serum	ExoQuick	NA	NA	C57BL/6J mice, RIP-CreER mice, and Rosa26-GNZ mice	Intraperitoneal and intravenous injections	Increased pancreatic beta-cell proliferation
[82]	Human bone marrow MSCs	Total exosome isolation reagent (Invitrogen)	NA	NA	NSG Mice (NOD scid gamma)	Intraperitoneal infusion	Inhibition of immune rejection
[81]	Endothelial progenitor cells isolated from PBMCs	UC	NA	FC, EM	Immunodeficient (SCID) Mice	Subcutaneous implantation	Enhancement of neo-angiogenesis of human pancreatic islets
[175]	Adipose-derived MSCs	UC	40–100 nm	FC, EM, DLS, SEM	6- to 8-week old C57BL/6 male mice	Intraperitoneal injection	Enhanced regulatory T-cell population without change in the proliferation index of lymphocytes
[110]	Human MSCs	UC	NA	NTA	NA	NA	MSC-derived MVs suppress inflammatory T-cell responses in the islet antigens through the promotion of regulatory dendritic cells in T1DM
[80]	Human islets	UC	54–256 nm	FC, EM, WB, SEM	NA	NA	Human islet-derived EVs participate in beta cell-endothelium cross-talk and the neoangiogenesis process
[163]	Menstrual blood-derived MSC	Exo-spin kit	30–150 µm	AFM, FE-SEM, WB	Male Wistar Rats (STZ-induced rat model of T1DMM)	Intravenous injection	EVs isolated from stem cells may regenerate beta-cells of islets through the Pdx-1 pathway

### 4.7. EV Based Clinical Trials in T1DM

One of the main reasons that EVs are a preferred option as therapeutic delivery vehicles is their ability to decrease the harmful effects that foreign compounds have on the body when introduced. Because of their biological origin, EVs are unlikely to elicit a strong immunological response. EVs are also safe because they are fully non-replicative and non-mutagenic; therefore, there are no regulatory considerations about side effects or the development of neoplasia [176]. The low toxicity of EVs in EV-based in vivo therapies has corroborated these benefits. However, there are a number of possible safety concerns. Further studies are warranted to assure that any individual application for EVs is safe. To date, there are now seven T1DM related clinical studies (Table 6) available by searching the clinicaltrials.gov database with keywords such as “Type 1 diabetes”, “extracellular vesicle”, “exosome”, or “microvesicle.” The majority of these clinical trials utilize EVs as biomarkers for clinical diagnosis and tracking disease progression post-treatments since EVs carry lipids, proteins, DNA, mRNAs, miRNAs, lipids, and proteins representative of their parent cell. Due to the ubiquitous use of MSC therapies, MSC EVs are used in the majority of clinical and preclinical investigations. Since MSC-derived EVs contain MSC-released secretomes such as cytokines, chemokines, and anti-inflammatory substances, they may have similar therapeutic potential [177]. This research, both clinical and in cell or animal models, on the use of EVs as biomarkers and therapeutics points to the great value EVs can add to the field of T1DM.

## 5. Conclusions

T1DM and its related complications have increasingly become a worldwide health problem and are associated with an enormous healthcare and economic burden. Patients diagnosed with type 1 diabetes often suffer from hypo- or hyper-glycemic episodes due to the cost and biological limitations of current therapeutic options. Transplants, in particular islet transplants, are long-lasting and impactful therapeutic alternatives to continuous exogenous insulin administration. However, before islet transplants can be regularly used, there are a number of complications that must be addressed, including the fact that up to 70% of transplanted islet cells may be destroyed during cell culture or after transplantation, with a very slow rate of self-replication [48,178]. This may be due to insults such as enzymatic damage, oxidative stress, and detachment of islet cells [78]. As such, future strategies for increased islet survival during transplantation include the generation of functional islet β-cells from easily replicating human or porcine pluripotent stem cells to allow for greater transplant numbers [48,78]. Alternative delivery techniques for islet cells such as encapsulation, scaffolds, or transplant sites may lead to less immune activation and improved long-term cell survival and function [72,78].

**Figure 4:**
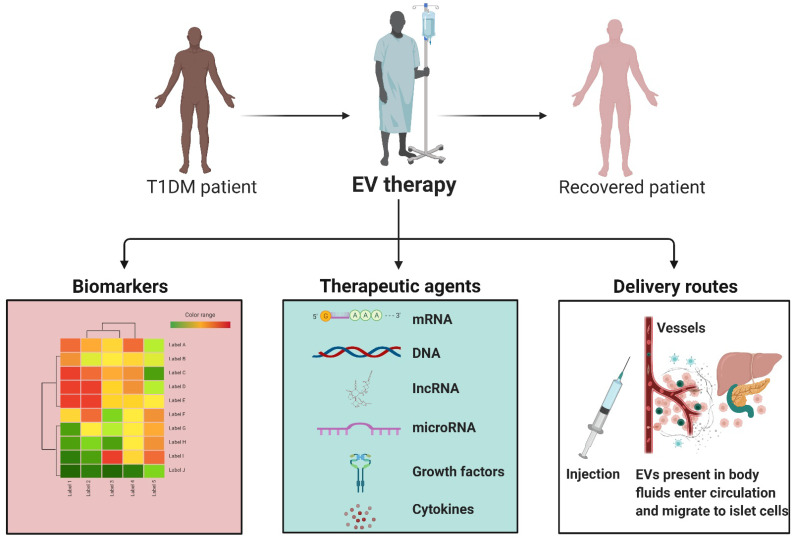
EVs in T1DM. EVs can play a valuable role in T1DM. There are a number of RNAs and proteins that are modified in EVs in T1DM and rejected transplants. In addition, the natural components of EVs from a healthy system, such as anti-inflammatory cytokines or micRNAs, can aid in regulating and correcting a disease state. Moreover, EVs can be loaded with particular cargos and delivered alongside transplantation to aid in graft survival and long-term well-being. EVs can be are delivered into the bloodstream where they migrate to distant tissues such as pancreatic islets of Langerhans and are engulfed by target cells. As the EV payload is delivered into a target cell, the proteins and RNA species can have various impacts, triggering diverse cell signaling cascades and the regulation of gene expression.

More work needs to be carried out to enhance biomarkers of T1DM, transplant rejection, and combined treatment options; EVs are a novel, versatile tool allowing new approaches for both biomarkers and therapeutics (Figure 4). EVs are mediators of intercellular communication, and cell-specific EVs carry disease-specific molecular signatures that allow the identification of T1DM from healthy patients, as well as serve as biomarkers of diabetic complications, including nephropathy and retinopathy [130,131,132,133,134]. Next, the focus should be on the identification of status-specific EV biomarkers for T1DM disease and transplant prognostic signature. Further, EVs are a pivotal strategy in ameliorating many of the complications associated with pancreas and islet transplantation outcomes [150]. However, additional investigations are needed regarding EV production, isolation, characterization, the underlying mechanism of actions, best route of administration, timing, and clearance patterns for a better understanding of T1DM therapeutic strategies [114,135,179,180].

## Figures and Tables

**Table 2:** Typical protein families found within EVs [38,39].

Protein Family	Protein(s)
MVB synthesis	HRS, Alix, TSG101, Clathrin
Tetraspanins	CD9, CD63, CD81, CD82, CD151, CD53, Peripherin-2, Uroplakin-1a, Uroplakin-1b
Signal transduction	Protein kinases, 14-3-3, G proteins, CBL, LCK
Heat shock proteins	Hsp70, Hsp90, HSPA5, Cyclophilin, Hsp84, TCP-1 chaperone family β-subunit TCP-1 chaperone family γ-subunit TCP-1 chaperone family ε-subunit
Cytoskeletal proteins	Actin, Tubulin, Moesin, Cofilin 1, Myosin
Membrane transport and fusion	Rabs, Annexins, GTPases, Flotillins, Annexin II Annexin V
Metabolic Enzymes	GAPDH, Enolase 1, PKM2, PGK1, Thioredoxin peroxidase, Malate dehydrogenase Fatty acid-binding protein-3 Fatty acid-binding protein-4
Lipid related proteins	Flotillin, Glycosylphos phatidylinositol anchored proteins
Antigen presentation	MHC I, MHC II, CD86
Adhesion	Integrins, MFGE8

**Table 3:** Characteristics of EV isolation methods (Adapted from [38]).

	Isolation Technique	Equipment	Isolation Principle	Advantages	Disadvantages	Yield	Purity
Traditional methods	Ultra-centrifugation	Ultra-centrifuge	Physical method	High sample capacity; Protein and RNA components are not affected; Facilitated later research	Time-consuming; Instrument-dependent	Low	Low
	Density gradient	Ultra-centrifuge	Physical method	High separation efficiency; EVs will not be crushed or deformed	Extended run time; Equipment dependence; Complex process	Low	High
	Immuno-magnetic beads	Magnetic bead, antibody	Chemical method	Time-efficient; Maintain integrity; Convenient operation; Not affected by EV size; No need for expensive instruments	High reagent cost; Low capacity	Low	High
	Precipitation	Ultra-centrifuge	Physical/Chemical method	High yield; Easy; Concentrates diluted samples	Post-clean up is needed for downstream applications	High	Low
Emerging methods	ExoQuick	ExoQuick kit	Physical/Chemical method	Simple steps, Quick operation; Size uniformity; Suitable for small samples, such as serum	Affected by EV diameter; Expensive reagents	High	Low
	Size Exclusion Chromatography	Gel filtration column	Physical/Chemical method	Uniform in size	Low extraction volume; Extensive laboratory equipment requirements	High	High
	Stirred ultrafiltration	Ultra-filtration membrane, Nitrogen gas	Physical method	Does not rely on equipment; Less time consuming than other methods; Reduces the destruction of EVs during the process	Loss of EV during the process	High	Moderate
	Filtration Device	Microfluidic devices (e.g., nano traps)	Physical/Chemical method	Fast, Low cost; Easy automation and integration; High portability	Lack of standardization and large-scale tests on clinical samples, Lack of method validation; Low sample capacity	High	Low
	nPES	GNPs, Antibodies	Chemical method	Fast; Efficient; Quantitative analysis	High reagent cost; Complex statistical tools; Low capacity	Low	High
	Membrane modification	Magnetic field, Magnetic nanoparticles	Physical/Chemical method	Needs no antibodies; Save time; preserve the original structure of the EVs; Drug carriers	Complicated operation	Low	Low
	ExoTIC	ExoTIC, Syringe, Pump	Physical/Chemicalmethod	Simple operation; EVs in aspecific range	Special equipment requirements; Lack of tests on clinical samples	High	High
	Flow field-flow fractionation	Flow field-flow fractionation instrument	Physical method	Label-free isolation; Large scale production	Special instrument requirement; Costly	High	High

**Table 6:** EV-based clinical trials in T1DM.

Study Title	EV Source	Administration Route/Test	Dose Reported	Study Identifier	Status
Importance in Type 1 Diabetes Patients of an Optimized Control of Post-Prandial Glycaemia on Oxidant Stress Prevention	Blood	Preprandial or postprandial injection	NA	NCT00934336	Complete
Treatment Effects of Atorvastatin on Hemostasis and Skin Microcirculation in Patients with Type 1 Diabetes	Blood	Tablets	80 mg once daily	NCT01497912	Complete
Circulating Extracellular Vesicles Released by Human Islets of Langerhans	Blood	NA	NA	NCT03106246	Recruiting
Characterization of Adult-Onset Autoimmune Diabetes	Blood	Mixed Meal Test	NA	NCT03971955	Recruiting
Insulin Deprivation on Brain Structure and Function in Humans with Type 1 Diabetes	Blood	NA	NA	NCT03392441	Recruiting
Development of Novel Biomarkers for the Early Diagnosis of Type 1 Diabetes	Blood	NA	NA	NCT04164966	Recruiting
Effect of Microvesicles and Exosomes Therapy on β-cell Mass in Type I Diabetes Mellitus	MSC	NA	Insulin, exosomes, and microvesicles	NCT02138331	NA

## Data Availability

Not applicable.

## Abbreviations

UC	ultracentrifugation
EM	electron microscopy
WB	western blot
NTA	nanoparticle tracking analysis
FC	flow cytometry
PBS	phosphate buffered saline
DLS	dynamic light scattering
SEM	scanning electron microscopy
